# Induced Local and Systemic Defense Responses in Tomato Underlying Interactions Between the Root-Knot Nematode *Meloidogyne incognita* and the Potato Aphid *Macrosiphum euphorbiae*

**DOI:** 10.3389/fpls.2021.632212

**Published:** 2021-04-14

**Authors:** Crispus M. Mbaluto, Esraa M. Ahmad, Anne Mädicke, Katharina Grosser, Nicole M. van Dam, Ainhoa Martínez-Medina

**Affiliations:** ^1^Molecular Interaction Ecology, German Centre for Integrative Biodiversity Research (iDiv) Halle-Jena-Leipzig, Leipzig, Germany; ^2^Institute of Biodiversity, Friedrich-Schiller-Universität-Jena, Jena, Germany; ^3^Department of Genetics, Faculty of Agriculture, Cairo University, Giza, Egypt; ^4^Plant-Microorganism Interaction, Institute of Natural Resources and Agrobiology of Salamanca (IRNASA-CSIC), Salamanca, Spain

**Keywords:** aboveground-belowground interactions, phytohormones, plant-mediated interactions, potato aphid (*Macrosiphum euphorbiae*), root-knot nematodes (*Meloidogyne incognita*), steroidal glycoalkaloids, systemic responses, local responses

## Abstract

Plants mediate interactions between different herbivores that attack simultaneously or sequentially aboveground (AG) and belowground (BG) organs. The local and systemic activation of hormonal signaling pathways and the concomitant accumulation of defense metabolites underlie such AG-BG interactions. The main plant-mediated mechanisms regulating these reciprocal interactions *via* local and systemic induced responses remain poorly understood. We investigated the impact of root infection by the root-knot nematode (RKN) *Meloidogyne incognita* at different stages of its infection cycle, on tomato leaf defense responses triggered by the potato aphid *Macrosiphum euphorbiae*. In addition, we analyzed the reverse impact of aphid leaf feeding on the root responses triggered by the RKN. We focused specifically on the signaling pathways regulated by the phytohormones jasmonic acid (JA), salicylic acid (SA), abscisic acid (ABA), and indole-3-acetic acid (IAA) as well as steroidal glycoalkaloids as induced defense compounds. We found that aphid feeding did not induce AG hormonal signaling, but it repressed steroidal glycoalkaloids related responses in leaves, specifically when feeding on plants in the vegetative stage. Root infection by the RKN impeded the aphid-triggered repression of the steroidal glycoalkaloids-related response AG. In roots, the RKN triggered the SA pathway during the entire infection cycle and the ABA pathway specifically during its reproduction stage. RKN infection also elicited the steroidal glycoalkaloids related gene expression, specifically when it was in the galling stage. Aphid feeding did not systemically alter the RKN-induced defense responses in roots. Our results point to an asymmetrical interaction between *M. incognita* and *Ma. euphorbiae* when co-occurring in tomato plants. Moreover, the RKN seems to determine the root defense response regardless of a later occurring attack by the potato aphid AG.

## Introduction

Plants encounter several species of insect herbivores and pathogens that reduce their fitness. To defend themselves against these attackers, plants have evolved multifaceted mechanisms to perceive and appropriately respond to the specific attackers, thus preventing or attenuating the attack (Mithöfer and Boland, 2008; War et al., 2012; Mortensen, 2013). Plant hormones regulate the plant’s immune system (Pieterse et al., 2012). Among them, jasmonic acid (JA) with its derivates (collectively called jasmonates; JAs) and salicylic acid (SA) are considered as major defense hormones (Pieterse et al., 2009, 2012; Erb et al., 2012). The activation of phytohormone related pathways occurs with considerable specificity. The JA pathway is typically (but not exclusively) activated upon the attack of chewing herbivores and necrotrophic pathogens, while piercing-sucking herbivores and biotrophic pathogens trigger the SA pathway (Walling, 2000; Zhu-Salzman et al., 2004; Howe and Jander, 2008; Diezel et al., 2009; Lemarié et al., 2015; Wasternack, 2015). While the JA and SA pathways form the backbone of the plant’s immune system, other hormones such as ethylene, abscisic acid (ABA), auxins, and cytokinins also contribute to defense signaling (Bari and Jones, 2009; Erb et al., 2012; Kammerhofer et al., 2015). These hormones can antagonistically or synergistically interact with the JA-SA backbone of the plant’s immune signaling network. This so-called hormone cross-talk provides the plant with a powerful capacity to finely regulate its immune response to the specific attacker (Pieterse et al., 2009; Li et al., 2019).

The induction of plant defense responses upon herbivory at local sites is often accompanied by systemic induced responses in distal tissues, thereby protecting undamaged plant parts from subsequent attack. Systemic signaling is not limited to the particular organ (roots or shoots) under attack, but it can cross the root-shoot interface. Several studies show that the attack by aboveground (AG) and belowground (BG) herbivores and pathogens leads to systemic responses mediated *via* the plant which influence organisms associated with the other organ. BG herbivores can induce systemic responses in AG plant parts that can facilitate or impede the performance of the AG insect herbivores. For example, root damage by the insect herbivore *Tecia solanivora* decreases the performance of the AG leaf chewers *Spodoptera exigua* and *Spodoptera frugiperda* when feeding on potato plants (Kumar et al., 2016). On the other side, root infection by the parasitic root nematode *Globodera pallida* increased the reproductive success of the AG-feeding aphid *Myzus persicae* (Hoysted et al., 2017). Although less studied, AG herbivory can also systemically influence the performance of herbivores feeding on BG plant parts. For example, simulated AG herbivory by *Manduca sexta* on *Nicotiana attenuata* enhanced the performance of the parasitic root nematode *Meloidogyne incognita* (Machado et al., 2018). In contrast, AG herbivory can also negatively affect BG-feeding herbivores. For example, leaf-feeding by *Spodoptera littoralis* on maize plants deterred larvae of *Diabrotica virgifera virgifera* from infesting the roots (Erb et al., 2015).

These AG-BG plant-mediated interactions are driven at least partially, by the activation of hormonal-related pathways triggered by the attacking herbivores. This leads to changes in plant primary metabolism as well as the production of compounds that are toxic or deterrent for the herbivores, at both local and systemic sites. For instance, root herbivory and root elicitation by exogenous application of JA result in the accumulation of secondary metabolites, including steroidal glycoalkaloids, glucosinolates, and nicotine in leaves of different plant species, including *N. attenuata*, *Solanum tuberosum*, and *Cardamine hirsuta* (Fragoso et al., 2014; Kumar et al., 2016; Bakhtiari et al., 2018). On the other hand, AG herbivory or elicitation by exogenous application of methyl jasmonate enhances JA biosynthesis and the accumulation of secondary metabolites, including steroidal glycoalkaloids, phenolic acids, and glucosinolates in roots of several plants, including tomato, potato, brussels sprouts, *Brassica nigra*, *Solanum dulcamara*, and maize (Hlywka et al., 1994; Soler et al., 2007; Abdelkareem et al., 2017; Calf et al., 2020; Karssemeijer et al., 2020; Mbaluto et al., 2020). Such systemically-triggered changes in plant defense compounds can drive the impact on the performance of herbivores in the opposite compartment (Kumar et al., 2016; Bakhtiari et al., 2018; van Dam et al., 2018; Karssemeijer et al., 2020).

Most of the studies addressing the systemic defense-responses elicited by AG and BG interacting attackers focus on insect herbivores. Moreover, the majority of these studies focus on the impact of one herbivore feeding on one organ (AG or BG) on the induced systemic responses and the effect of the herbivores feeding on the other organ (Erb et al., 2009; Kumar et al., 2016; Hoysted et al., 2017, 2018; Machado et al., 2018; van Dam et al., 2018; Karssemeijer et al., 2020). It remains less explored how plants integrate sequential BG and AG attack, and the resulting concomitant induced responses in AG and BG organs of the same plant (Kutyniok and Muller, 2012; McCarville et al., 2012; Kammerhofer et al., 2015). In this study, we analyzed the systemic induced defense responses underlying the reciprocal interaction between root-knot nematodes (RKNs) and AG-feeding aphids sharing the same host.

Root-knot nematodes are soil-inhabiting parasites that infect the roots of thousands of plants. As obligate root feeders, they spend most of their life inside roots, thereby significantly influencing root physiology. After egg hatching, the infective second-stage juveniles (J2s) penetrate their host roots and migrate in between cells to reach and settle in the vascular cylinder (Fenoll et al., 1997; Perry et al., 2009). They select several vascular cells to induce their feeding sites, commonly known as giant cells. As they feed and develop further to reach maturity, they secrete and inject effector molecules that cause hyperplasia and hypertrophy of cells surrounding the giant cells to form root galls. Throughout the development, RKNs manipulate the host’s phytohormonal signaling in order to suppress defense responses and establish a sink for nutrients (Gheysen and Mitchum, 2011, 2019). Remarkably, several studies demonstrate that root infection by RKNs also affects defense-related responses in AG plant parts (Hamamouch et al., 2011; Arce et al., 2017; van Dam et al., 2018). However, the studies dealing with the impact of RKNs on AG defenses are scarce and show contrasting results. For instance, in *Arabidopsis thaliana* and rice plants, root infection by different RKN species was found to both increase and decrease systemically in leaves the JA levels and the expression of marker genes in the JA and SA pathways, depending on the study systems (Hamamouch et al., 2011; Kyndt et al., 2017b).

Aphids are insect herbivores that in analogy to RKNs, feed directly on vascular content. They insert their mouthparts (stylet) in between the primary and secondary cells layers of the leaf to reach the sieve elements in the vascular tissues. Plants generally respond to aphid attack by activating the SA responsive pathway (Walling, 2000; De Vos et al., 2005), although some studies revealed the activation of the JA pathway upon aphids attack (Fidantsef et al., 1999) as well as the negative impact of JA elicitation on aphids feeding (Cooper and Goggin, 2005). Remarkably, it has been shown that aphids can trigger systemic induction of defenses in roots, and influence BG-associated biota. For example, AG herbivory by the aphid *Brevicoryne brassicae* triggered an increase in JA levels systemically in roots of *Brassica oleracea*, although this increase did not affect the development of root fly *Delia radicum* (Karssemeijer et al., 2020).

Because plant-parasitic root nematodes and aphids tap resources from the vascular tissues, they can affect each other *via* direct competition or by systemically triggering the plant’s defense system (Hol et al., 2013). In this study, we aimed to disentangle the molecular and chemical mechanisms driving the plant-mediated reciprocal interaction between RKNs and AG feeding aphids. With this aim, we established a bioassay including the important crop species tomato (*Solanum lycopersicum*) and addressed the effects of root infection by the RKN *Ma. incognita* on leaf defenses triggered by the potato aphid *Macrosiphum euphorbiae*; as well as the impact of leaf herbivory by *Ma. euphorbiae* on root defenses induced by *M. incognita*. Because the interactions between AG herbivores and *M. incognita*-induced plant defense responses are modulated by the RKN infection stages (Mbaluto et al., 2020), we studied the *M. incognita*-*Ma. euphorbiae* interaction during the different stages of the *M. incognita* infection cycle namely; invasion, galling, and reproduction. Our results show that *M. incognita* has a moderate systemic effect on defense responses triggered locally in leaves by *Ma. euphorbiae*. Our results further indicate that this systemic effect is modulated during the *M. incognita* root infection cycle. On the other hand, *Ma. euphorbiae* did not interfere systemically with the defense responses triggered by *M. incognita* locally in roots. Our findings suggest an asymmetrical interaction between *M. incognita* and *Ma. euphorbiae* when co-occurring in tomato plants, where *M. incognita* seems to determine the root defense response regardless of the AG *Ma. euphorbiae* attack.

## Materials and Methods

### Nematode and Aphid Cultures

We used the RKN *M. incognita* as the BG herbivore and the potato aphid *Ma. euphorbiae* as the AG herbivore. The *M. incognita* colony was initially obtained from Rijk Zwaan (De Lier, Netherlands) and maintained on tomato cv “Moneymaker” in a glasshouse. The colony was initiated from a single egg mass, and 8 weeks later, eggs were extracted for use in the experiments (Martínez-Medina et al., 2017). The potato aphid was kindly provided by Dr. Zeger van Herwijnen (Rijk Zwaan Breeding B.V De Lier, Netherlands). We maintained a laboratory colony using the leaf disc method (Rocca and Messelink, 2017) with slight modifications. In brief, we prepared 1% (w/v) water-agar and poured in plastic boxes 8 cm (length) × 5 cm (width) × 4 cm (height) to obtain ~0.5 cm thickness. A leaf disc from *Capsicum annuum* was embedded on the solidified agar with the abaxial side facing up to mimic normal aphid feeding side or position. The colony was maintained in a growth chamber (CLF PlantClimatic, CLF PlantClimatics GmbH, Wertingen, Germany) under 12-h light, 22°C: 12-h dark, 20°C, 45% relative humidity conditions. In the bioassays, we used apterous individuals.

### Plant Material and Growth Conditions

We used tomato (*S. lycopersicum*) cultivar “Moneymaker,” as the study model plant. Tomato seeds were obtained from Intratuin B.V (Woerden, Netherlands). The seeds were sterilized, germinated, and transplanted, according to Mbaluto et al. (2020). In the glasshouse, the plants were randomly distributed and grown under 16-h light 25 ± 3°C: 8-h dark 22 ± 3, 40% relative humidity conditions. The plants were watered as required and supplemented weekly with half-strength Hoagland solution (Hoagland and Arnon, 1938). Four weeks after germination, we used the plants for the bioassays.

### Nematode Inoculation and Aphid Infestation

In order to mimic the natural sequence of events, we infected plants with the RKN *M. incognita* first. Indeed, root feeders such as plant-parasitic nematodes are among the first pests encountered by annual plants; while AG feeders such as aphids generally arrive later in the plant’s life cycle (Bezemer and van Dam, 2005; van Dam et al., 2018). In all the bioassays, the plants assigned for *M. incognita* inoculation received 3,000 *M. incognita* eggs suspended in 1 ml of tap water (Mbaluto et al., 2020). Plants not assigned for *M. incognita* inoculation were mock-inoculated with 1 ml of tap water. We established three-time points after the *M. incognita* inoculation, corresponding to the main stages of its life cycle: 5 days post nematode inoculation (dpi), corresponding to the invasion stage; 15 dpi corresponding to the galling stage, and 30 dpi corresponding with the reproduction stage (Mbaluto et al., 2020). At each specific time point after *M. incognita* inoculation, plants assigned to the AG herbivore were challenged with 12 *Ma. euphorbiae* individuals of mixed-stages (adults and nymphs). The aphids were contained on a single leaf for 24 h, using a round clip cage of 7 cm in diameter. The clip cage was mounted on one fully expanded leaf: specifically on the three leaflets close to the tip (Bandoly and Steppuhn, 2016). Similarly, we mounted an empty clip cage on similar leaves, as mentioned above, on the plants not assigned for the aphid infestation. At each study time point, i.e., invasion, galling, and reproduction stage, we established four treatment groups including; (1) controls: plants not challenged with any of the herbivores, (2) plants root-infected with *M. incognita*, (3) plants infested on leaves with *Ma. euphorbiae*, and (4) plants infected with *M. incognita* in roots and infested with *Ma. euphorbiae* on leaves. Ten biological replicates of each treatment per time point were established, giving a total of 120 plants. At 24 h after infesting the plants with aphids, we harvested the plants, starting with the leaves and followed with the roots samples. We selected this specific time point (i.e., 24 h) in accordance with previous studies (Kafle et al., 2017), and also based on a pilot experiment in which the time points 12, 24, and 48 h were tested (data not shown). For root sampling, the entire root system was harvested. For the leaves, we harvested specifically the leaves that the aphids were feeding on, or the leaves that were mounted with empty clip cages without aphids, in the case of non-infested plants. Leaf and root material was stored at −80°C until use. In addition, after washing the root systems, we counted the number of galls visible to the naked eye from the root system of *M. incognita* infected plants. Approximately, the number of galls visible at the galling stage (15 dpi) averaged between 120 and 130 per plant. The number of visible galls had increased to 280–300 per plant at the reproduction stage (30 dpi).

### Assessment of the Impact of Nematode Root Infection on Aphids Performance

We assessed the impact of *M. incognita* root infection on the reproduction of *Ma. euphorbiae* by comparing the number of nymphs produced by the aphids on tomato plants that were root infected or not with *M. incognita*. For this, we established a bioassay in which we inoculated tomato plants with *M. incognita* eggs as described above. Plants not assigned for *M. incognita* inoculation were mock-inoculated with 1 ml of tap water. We established three-time points after the *M. incognita* inoculation, coinciding with the invasion, galling, and reproduction stages of *M. incognita*, as described above. At each specific time point after *M. incognita* inoculation, we carefully placed three apterous *Ma. euphorbiae* adults using a soft-bristled brush on a similar leaf to the one used in the defense response experiments. We allowed the adult female aphids to feed on the plants and reproduce for 3 days, after which we counted the number of nymphs on the third day. This experiment was conducted twice, with similar results.

### Phytohormone Extraction and Analysis

We extracted and quantified phytohormones from 100 mg of leaf and root material following the protocol previously described by Machado et al. (2013), with slight modifications. The extraction solution contained deuterated form of each phytohormone as the internal standards (i.e., *D*6-JA, *D*6-JA-*Ile*, *D*6-ABA, *D*5-IAA, and *D*6-SA). At the nebulization stage, the compounds were nebulized by electron spray ionization in the negative mode using the following conditions: capillary voltage 4,500 eV, cone gas 35 arbitrary units/350°C, probe gas 60 arbitrary units/300°C, and nebulizing gas at 60 arbitrary units. Data acquisition and processing were performed using the “MS data Review” software (Bruker MS Workstation, version 8.2, Bruker, Bremen, Germany). Phytohormone levels were calculated based on the peak area of the corresponding internal standard and the amount of fresh mass of plant material (ng^−1^ mg^−1^ FW), according to Mbaluto et al. (2020).

### Real-Time Quantitative qPCR

Total RNA was extracted from ~100 mg (fresh weight) of ground leaf and root material, according to Oñate-Sánchez and Vicente-Carbajosa (2008). We performed quality check both quantitative and qualitative using a NanoPhotometer® P330 (Implen, Munich Germany) and by gel electrophoresis (1% agarose). We removed traces of DNA by treating 5 μg of the extracted RNA with 2 U/μl of DNaseI (Thermo Fisher Scientific, Schwerte, Germany) and following the manufacturer’s instructions. The clean RNA was rechecked for quality as stated above. We synthesized the first-strand cDNA from 1 μg DNase free RNA by reverse transcription using 200 U/μl Revert Aid H-minus RT (Thermo Fisher Scientific Baltic UAB, Vilnius, Lithuania) following the manufacturer instructions. The amplification cycle conditions for cDNA synthesis were: at 42°C for 60 min, 50°C for 15 min, and 70°C for 15 min using a Thermal cycler (Techne, Stone, United Kingdom). Real-time quantitative qPCR reactions and relative quantification of specific *m*RNA levels were performed using CFX 384 Real-Time PCR system (Bio-Rad Laboratories Inc., Singapore), and with gene-specific primers described in Supplementary Table 1. The RT-qPCR cycle conditions were: 2 min at 50°C, 2 min at 95°C, and 40 cycle of 15 s at 95°C, and 60 s at 60°C (Vos et al., 2015). Melting curves analysis was done to verify the amplification of each gene transcript. Three technical replicates of each sample were included in the RT-qPCR. The gene expression levels were determined by normalizing the data to the reference gene *SIEF* (X14449), which encodes for the tomato elongation factor 1α (Miranda et al., 2013; Martínez-Medina et al., 2017). The stability of the *SIEF* gene was previously evaluated in the different tissues (roots and leaves) and under the different experimental conditions (nematode and aphids challenge) analyzed here. Normalized gene expression data were analyzed by the 2^−∆∆ct^ method (Livak and Schmittgen, 2001).

### Extraction of Metabolites and Data Processing

We extracted ~100 mg fresh leaf and root material for metabolites analysis following the method described by Mbaluto et al. (2020) with slight modifications. In brief, the modifications included, using formic acid in methanol (0.05% v/v) as solvent B in the mobile phase. The separation and characterization of secondary metabolites were done according to Mbaluto et al. (2020). The data was processed in MS-DIAL (v. 4.00, RIKEN) according to Moreno-Pedraza et al. (2019) and with modification of several parameters including retention time-end = 12.5 min, mass range end = 1,500 mass to charge ratio (*m/z*), and the alignment parameter setting: retention time tolerance = 0.2 min. We generated two datasets (i.e., leaves and roots datasets) from which we selected all features with mass to charge ratio (*m/z*) of 576.3 and 578.4 at retention time 11–12 min for each study time point. These *m/z* values represent fragments of the main steroidal glycoalkaloids in tomato α-dehydrotomatine and α-tomatine (Cataldi et al., 2005).

### Statistical Analysis

Datasets were analyzed using R software v 3.6.1 (R Core Development Team, 2019) unless indicated otherwise. Following three-way ANOVAs with factors *M. incognita* (Mi), *Ma. euphorbiae* (Me), and time (T) as model explanatory factors; two-way ANOVAs with *M. incognita* (Mi) and *Ma. euphorbiae* (Me) as factors were performed for each time point [invasion (5 dpi), galling (15 dpi), and reproduction (30 dpi) stages] to analyze the impact of plant herbivory on the phytohormones, steroidal glycoalkaloids, and the gene expression. Before the ANOVA analysis, all data sets were pre-screened for outliers using the interquartile range (IQR) method as a function in R. The number of outlying values varied between treatment groups from 0 to 2. In cases where the ANOVA results were significant, we detected the differences between the treatment groups using Tukey’s Honest Significant Difference (HSD) for multiple comparisons at *p* ≤ 0.05.

## Results

### Impact of *Meloidogyne incognita* Root Infection on Leaf Hormonal Responses Triggered by *Macrosiphum euphorbiae* Feeding

**Figure 1:**
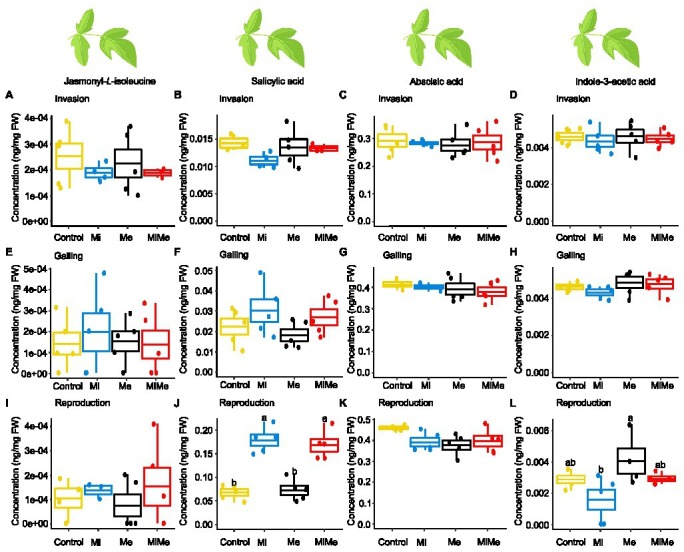
Phytohormones concentrations in tomato leaves upon aboveground and belowground herbivory. Mean concentrations (ng/mg fresh weight) of phytohormones in leaves of tomato plants infected belowground with *Meloidogyne incognita* (Mi), or infested aboveground with *Macrosiphum euphorbiae* (Me) or with both herbivores (MiMe). Control = plants without herbivores. Boxplots indicate the mean (±SEM, *n* = 5) concentrations of jasmonyl-*L*-isoleucine **(A,E,I)**, salicylic acid **(B,F,J)**, abscisic acid **(C,G,K),** and indole-3-acetic acid **(D,H,L)** measured at the nematodes’ invasion **(A-D)**, galling **(E-H)**, or reproduction **(I-L)** stages. Different letters above the boxplots indicate significant differences (*p* ≤ 0.05) in mean values between treatments, determined by Tukey's HSD test after ANOVA analysis.

**Figure 2:**
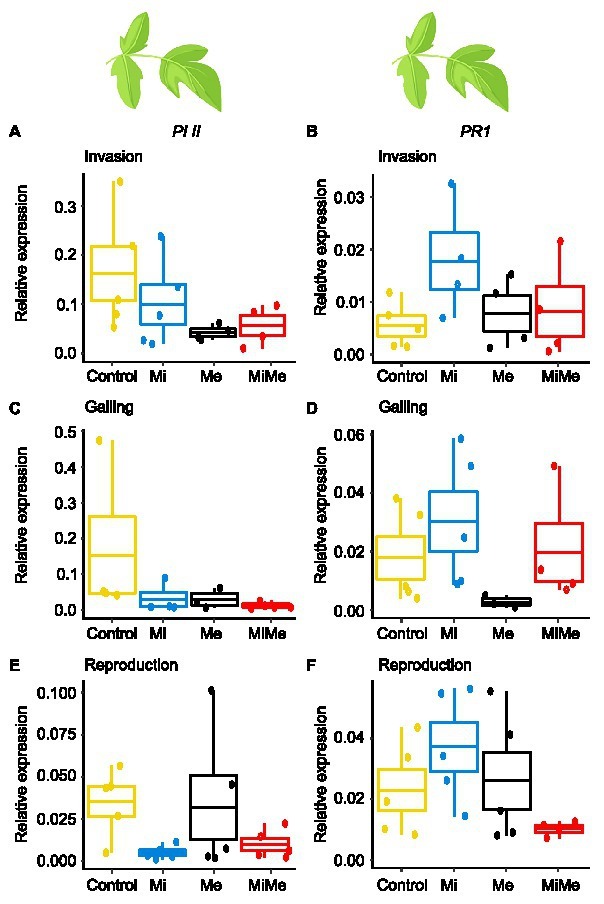
Expression of the jasmonic acid (JA) marker gene *Proteinase inhibitor II* (*PI II*) and the salicylic acid (SA) marker gene *Pathogenesis-related protein 1* (PR1) in tomato leaves upon aboveground and belowground herbivory. Relative expression of *PI II* and *PR1* genes were analyzed in leaves of tomato plants infected belowground with *Meloidogyne incognita* (Mi), or infested aboveground with *Macrosiphum euphorbiae* (Me) or with both herbivores (MiMe). Control = plants without herbivores. Boxplots indicate mean (±SEM, *n* = 5) expression values of *PI II* **(A,C,E)** and *PR1* **(B,D,F)**, measured at the nematodes’ invasion **(A,B)**, galling **(C,D)**, or reproduction **(E,F)** stages.

We first analyzed the local effect of *Ma. euphorbiae* herbivory on leaf hormonal responses when feeding on plants not inoculated with *M. incognita*. Because we used the *M. incognita* infection cycle stages [i.e., invasion (5 dpi), galling (15 dpi), and reproduction (30 dpi)] to time the experiment, the plants had different ages over the course of the experiment. This means that plants (young) infected by *M. incognita* as well as their respective control plants were 33 days-old when the *M. incognita* were at the invasion stage, those used when *M. incognita* were at the galling stage were 43 days-old (medium), and by the time the *M. incognita* had reached the reproduction stage, the plants were 58 days-old. We found that *Ma. euphorbiae* feeding did not alter the concentrations of JA-*Ile*, SA, and ABA compared to the control plants, regardless of plant age (Figures 1A–C,E–G,I–K; black vs. yellow boxplots; Supplementary Tables 2 and 8; JA levels were below the detection threshold). In agreement with the phytohormonal responses, *Ma. euphorbiae* feeding did not change the expression of *Proteinase inhibitor II* (*PI II*) and *Pathogenesis-related protein 1* (*PR1*) compared to controls (Figure 2; black vs. yellow boxplots; Supplementary Tables 3 and 9). Remarkably, *Ma. euphorbiae* feeding on 8 weeks old plants increased the overall levels of IAA (main effect of Me; Supplementary Table 8, *p* = 0.024), but there was no significant difference when compared with control plants (Figures 1D,H,L; black vs. yellow boxplots).

Next, we studied the systemic impact of *M. incognita* root infection, throughout its infection cycle, on leaf hormonal responses. *Meloidogyne incognita* root infection did not significantly affect the concentrations of JA-*Ile*, ABA, or IAA in tomato leaves compared to control plants at either of its infection cycle stages (Figures 1A,C–E,G–I,K,L; blue vs. yellow boxplots; Supplementary Tables 2 and 8). Despite a lack of JA-*Ile* response, *M. incognita* root infection overall downregulated the expression of the JA-responsive gene *PI II* compared to controls, as shown by a significant main effect (Supplementary Table 9, *p* = 0.028) in the reproduction stage (Figures 2A,C,E; blue vs. yellow boxplots; Supplementary Tables 3 and 9). Root infection by *M. incognita* significantly increased systemic SA levels compared to controls, specifically at the reproduction stage (Figure 1J; blue vs. yellow boxplot; Supplementary Tables 2 and 8). In contrast, the expression level of the SA-marker gene *PR1* in *M. incognita*-infected plants was not significantly different from that observed in controls (Figures 2B,D,F; blue vs. yellow boxplots; Supplementary Tables 3 and 9).

To decipher the systemic effect of *M. incognita* root infection on AG phytohormonal-related responses triggered by *Ma. euphorbiae* leaf herbivory, we compared plants challenged by both *M. incognita* and *Ma. euphorbiae* to those challenged with *Ma. euphorbiae* alone at each stage of *M. incognita* root infection cycle [Figures 1, 2, red (MiMe) vs. black (Me) boxplots; Supplementary Tables 2, 3, 8, and 9]. The levels of JA-*Ile*, ABA, and IAA, as well as the expression levels of *PI II* and *PR1* in leaves of co-infected plants were not significantly different from those infested with *Ma. euphorbiae* alone (Figures 1A,C–E,G–I,K,L, 2; red vs. black boxplots; Supplementary Tables 2, 3, 8, and 9). The SA levels were similar in *Ma. euphorbiae* and co-infected plants when *M. incognita* was at the invasion and galling stages (Figures 1B,F). Remarkably, when *M. incognita* was at the reproduction stage, the SA levels in co-infected plants increased compared to plants infested with *Ma. euphorbiae* alone (Figure 1J; red vs. black boxplots; Supplementary Tables 2 and 8).

### Effect of *Meloidogyne incognita* Root Infection on Leaf Accumulation of Steroidal Glycoalkaloids Induced by *Macrosiphum euphorbiae* Feeding

**Figure 3:**
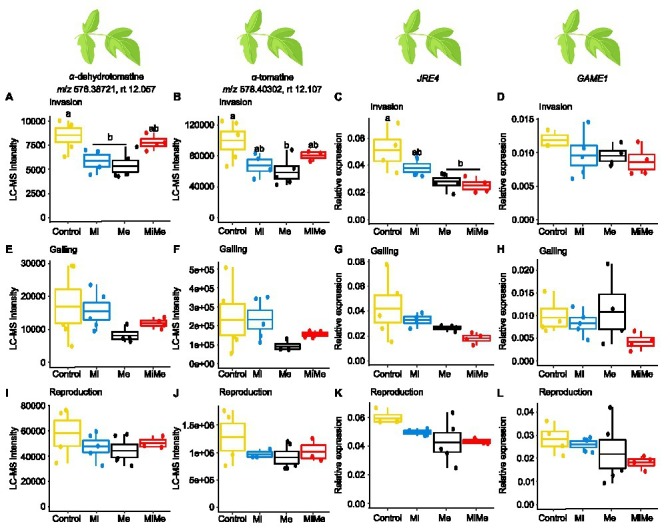
Relative intensities of the *m/z* signals of the steroidal glycoalkaloids α-dehydrotomatine and α-tomatine and relative expression of glycoalkaloid-related metabolism genes *jasmonate-responsive ethylene response factor 4* (*JRE4*) and *glycoalkaloid metabolism 1* (*GAME1*) in tomato leaves upon aboveground and belowground herbivory. Mean LC-MS intensities of α-dehydrotomatine (*m/z* 576.38721; rt. 12.057 min) and α-tomatine (*m/z* 578.40302; rt. 12.107 min) and expression of *JRE4* and *GAME1* in leaves tomato plants infected belowground with *Meloidogyne incognita* (Mi), or infested aboveground with *Macrosiphum euphorbiae* (Me) or with both herbivores (MiMe). Control = plants without herbivores. Boxplots are the mean (±SEM, *n* = 5) of α-dehydrotomatine **(A,E,I)**, α-tomatine **(B,F,J)**, *JRE4* **(C,G,K)**, and *GAME1* **(D,H,L)** measured at the nematodes’ invasion **(A-D)**, galling **(E-H)**, or reproduction **(I-L)** stages. Different letters above the boxplots indicate significant (*p* ≤ 0.05) differences in mean values between treatments, determined by Tukey's HSD test after ANOVA analysis.

Steroidal glycoalkaloids are important antiherbivore defense compounds in *Solanaceae* plants (Chowański et al., 2016). We first assessed the local effect of *Ma. euphorbiae* on leaf concentrations of the steroidal glycoalkaloids α-dehydrotomatine and α-tomatine, and the expression of the steroidal glycoalkaloid metabolism-related genes *jasmonate-responsive ethylene response factor (ERF) 4 transcription factor (JRE4;* encoding a master transcriptional regulator in defense-related steroidal glycoalkaloids) and *glycoalkaloid metabolism 1* (*GAME1*; encoding a UDP-Gal:tomatidine galactosyltransferase biosynthetic gene) when feeding on plants of different ages (Figure 3). Leaf feeding by *Ma. euphorbiae* led to a decrease in the concentrations of α-dehydrotomatine and α-tomatine, in young plants compared to controls (Figures 3A,B; black vs. yellow boxplots; Supplementary Tables 4 and 10). In agreement, *Ma. euphorbiae* feeding on young plants significantly downregulated the expression of *JRE4* (Figure 3C; black vs. yellow boxplot; Supplementary Tables 4 and 10). However, *Ma. euphorbiae* feeding did not affect *GAME1* expression in young plants (Figure 3D; black vs. yellow boxplot; Supplementary Tables 4 and 10). In the medium age and old plants, infestation by *Ma. euphorbiae* did not significantly alter the concentrations of α-dehydrotomatine and α-tomatine nor the expression of *JRE4* and *GAME1* compared to controls (Figures 3E–L; black vs. yellow boxplots; Supplementary Tables 4 and 10). These findings show that *Ma. euphorbiae* represses the accumulation of α-dehydrotomatine and α-tomatine in tomato leaves, specifically when feeding on plants in the vegetative stage.

We next studied the systemic impact of *M. incognita* root infection throughout its infection cycle on the leaf concentration of α-dehydrotomatine and α-tomatine as well as on the expression levels of *JRE4* and *GAME1* genes. At the invasion stage, the leaf concentration of α-dehydrotomatine and α-tomatine decreased in *M. incognita* infected plants compared to control plants (Figures 3A,B; blue vs. yellow boxplots; Supplementary Tables 4 and 10). Although not statistically significant (Main effect of Mi in Supplementary Table 10, *p* = 0.089), we found a slight downregulation of *JRE4* in leaves of plants that were infected with *M. incognita* at the invasion stage (Figure 3C; blue vs. yellow boxplot; Supplementary Tables 4 and 10). *Meloidogyne incognita* at the invasion stage did not affect the expression of the *GAME1* gene in leaves compared to control plants (Figure 3D, blue vs. yellow boxplot; Supplementary Tables 4 and 10). At the *M. incognita* galling and reproduction stages, the leaf levels of α-dehydrotomatine and α-tomatine, as well as the expression of *JRE4* and *GAME1*, were similar in *M. incognita* infected plants and control plants (Figures 3E–L; blue vs. yellow boxplots; Supplementary Tables 4 and 10). These results indicate that *M. incognita* triggers early and transient repression of the accumulation of α-dehydrotomatine and α-tomatine, specifically during the root invasion stage.

To check whether *M. incognita* root infection alters the repression of steroidal glycoalkaloid levels induced by *Ma. euphorbiae* in young (vegetative) plants, we compared plants challenged with *Ma. euphorbiae* alone to those co-infected with both *M. incognita* and *Ma. euphorbiae* at each of the *M. incognita* root infection cycle stages [Figure 3; red (MiMe) vs. black (Me) boxplots; Supplementary Tables 4 and 10]. At the invasion stage, co-infected plants had overall higher α-dehydrotomatine and α-tomatine levels than plants infested by *Ma. euphorbiae* alone (Figures 3A,B; red vs. black boxplots; Supplementary Tables 4 and 10 the interactive effect Mi^∗^Me). The expression of *JRE4* and *GAME1* in co-infected plants at the nematodes’ invasion stage was at a similar level to that in plants challenged with *Ma. euphorbiae* alone (Figures 3C,D; red vs. black boxplots; Supplementary Tables 4 and 10). At the galling and reproduction stages of *M. incognita*, the concentrations of α-dehydrotomatine and α-tomatine, as well as the expression of *JRE4* and *GAME1* in co-infected plants, were similar to those plants challenged with *Ma. euphorbiae* alone (Figures 3E–L; red vs. black boxplots; Supplementary Table 10). Our results indicate a moderate effect of *M. incognita* root infection on leaf steroidal glycoalkaloids associated with *Ma. euphorbiae* feeding.

### Impact of *Macrosiphum euphorbiae* Leaf Feeding on Root Hormonal Related Responses Triggered by *Meloidogyne incognita* Infection

**Figure 4:**
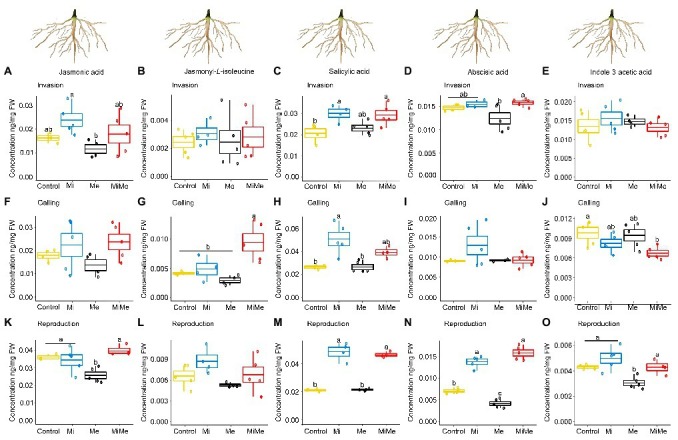
Phytohormones concentrations in tomato roots upon aboveground and belowground herbivory. Mean concentrations (ng/mg fresh weight) of phytohormones in roots of tomato infected belowground with *Meloidogyne incognita* (Mi), or infested aboveground with *Macrosiphum euphorbiae* (Me) or with both herbivores (MiMe). Control = plants without herbivores. Boxplots indicate the mean (±SEM, *n* = 5) of JA **(A,F,K)**, jasmonyl-*L*-isoleucine **(B,G,L)**, SA **(C,H,M)**; abscisic acid **(D,I,N)** and indole-3-acetic acid **(E,J,O)** concentrations measured at the nematodes’ invasion **(A-E)**, galling **(F-J)**, or reproduction **(K-O)** stages. Different letters above the boxplots indicate significant differences (*p* ≤ 0.05) in mean values between treatments, determined by Tukey's HSD test after ANOVA analysis.

**Figure 5:**
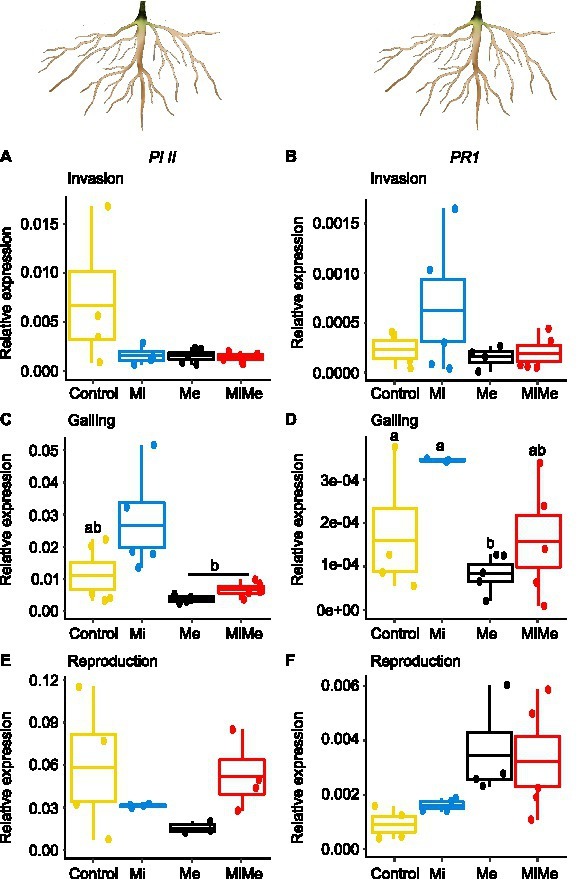
Expression of the jasmonic acid (JA) marker gene *Proteinase inhibitor II (PI II)* and the salicylic acid (SA) marker gene *Pathogenesis-related protein 1 (PR1)* in tomato roots upon aboveground and belowground herbivory. Relative expression of *PI II* and *PR1* genes were analyzed in roots of tomato plants infected belowground with *Meloidogyne incognita* (Mi), infested aboveground with *Macrosiphum euphorbiae* (Me), or with both herbivores (MiMe). Control = plants without herbivores. Boxplots indicate mean (±SEM, *n* = 5) expression values of *PI II* **(A,C,E)** and *PR1* **(B,D,F)** measured at the nematodes’ invasion **(A,B)**, galling **(C,D)**, or reproduction **(E,F)** stages. Different letters above the boxplots indicate significant differences (*p* ≤ 0.05) in mean expression among treatments, determined by Tukey's HSD test after ANOVA analysis.

We first analyzed the local impact of *M. incognita* infection on root phytohormonal-related responses throughout its infection cycle. *Meloidogyne incognita* root infection did not significantly affect the level of JA, JA-*Ile*, or IAA in tomato roots compared to controls and regardless of the infection cycle stage (Figures 4A,B,E–G,J–L,O; blue vs. yellow boxplots; Supplementary Tables 5 and 11). *Meloidogyne incognita* infection did not affect the expression of *PI II* compared to controls, regardless of the infection cycle stage (Figures 5A,C,E; blue vs. yellow boxplots; Supplementary Tables 6 and 12). *Meloidogyne incognita* root infection triggered the root accumulation of SA at all infection stages when compared to controls (Figures 4C,H,M; blue vs. yellow boxplots; Supplementary Tables 5 and 11). However, *M. incognita* root infection did not alter *PR1* expression compared to controls (Figures 5B,D,F; blue vs. yellow boxplots; Supplementary Tables 6 and 12). We found no differences in the levels of ABA in roots *M. incognita*-infected plants compared to controls when *M. incognita* was at the invasion and galling stages (Figures 4D,I; blue vs. yellow boxplots). However, at the reproduction stage, *M. incognita* significantly increased the ABA levels compared to control plants (Figure 4N; blue vs. yellow boxplot; Supplementary Tables 5 and 11).

Leaf herbivory by *Ma. euphorbiae* did not systemically affect root levels of JA-*Ile* or SA regardless of the plant age (Figures 4B,C,G,H,L,M; black vs. yellow boxplots; Supplementary Tables 5 and 11). We observed that JA, ABA, or IAA also did not systemically change when *Ma. euphorbiae* was feeding on young or medium-age (vegetative) plants (Figures 4A,D–F,I,J; black vs. yellow boxplots; Supplementary Tables 5 and 11). However, in old plants, *Ma. euphorbiae* feeding led to a significant decrease in the root levels of JA, ABA, and IAA (Figures 4K,N,O; black vs. yellow boxplots; Supplementary Tables 5 and 11). *Macrosiphum euphorbiae* feeding, in general, did not affect the expression level of *PI II* and *PR1*, regardless of plant age (Figure 5). Only in medium-aged plants, the expression levels of *PR1* decreased in the roots of plants challenged with *Ma. euphorbiae* (Figure 5D; black vs. yellow boxplot; Supplementary Tables 6 and 12).

We finally assessed whether *M. euphoribae* feeding affected the phytohormonal root responses associated with *M. incognita* root infection [Figures 4, 5; red (MiMe) vs. blue (Mi) boxplots; Supplementary Tables 5, 6, 11, and 12]. Roots infected by *M. incognita* alone had similar levels of JA, SA, ABA, and IAA as roots of plants co-infected with *M. incognita* and *Ma. euphorbiae* (Figures 4A,C–F,H–K,M–O; red vs. blue boxplots; Supplementary Tables 5 and 11). Similar to the SA levels, the expression of *PR1* in *M. incognita*-infected roots did not differ from that in roots of co-infected plants (Figures 5B,D,F; red vs. blue boxplots; Supplementary Tables 6 and 12). The levels of JA-*Ile* in the roots of co-infected plants were similar to that on roots of plants infected with *M. incognita* alone at the invasion and reproduction stages (Figures 4B,L). However, when *M. incognita* was at the galling stage, a higher level of JA-*Ile* was observed in the root of co-infected plants compared to roots of plants infected with *M. incognita* alone (Figure 4G; red vs. blue boxplot; Supplementary Tables 5 and 11). By contrast, a higher expression level of *PI II* was found in the roots of *M. incognita*-infected plants compared to expression in co-infected plants at the galling stage. At the invasion and reproduction stages, *PI II* expression was similar in *M. incognita* and co-infected plants (Figures 5A,E; red vs. blue boxplots; Supplementary Tables 6 and 12).

### Effect of *Macrosiphum euphorbiae* Leaf Feeding on Root Steroidal Glycoalkaloids Induced by *Meloidogyne incognita* Infection

**Figure 6:**
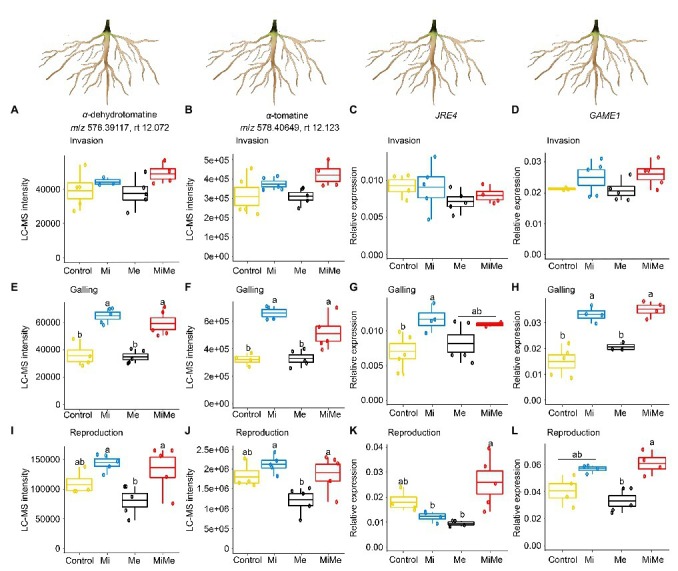
Relative intensities of the *m/z* signals of the steroidal glycoalkaloids α-dehydrotomatine and α-tomatine and relative expression of glycoalkaloid-related metabolism genes *jasmonate-responsive ethylene response factor (ERF) 4 transcription factor (JRE4)* and *glycoalkaloid metabolism 1 (GAME1)* in tomato roots upon aboveground and belowground herbivory. Mean LC-MS intensities of α-dehydrotomatine (*m/z* 576.39117; rt. 12.072 min) and α-tomatine (*m/z* 578.40649; rt. 12.123 min) and expression of *JRE4* and *GAME1* in roots of tomato plants infected belowground with *Meloidogyne incognita* (Mi), infested aboveground with *Macrosiphum euphorbiae* (Me), or with both herbivores (MiMe). Control = plants without herbivores. Boxplots indicate the mean (±SEM, *n* = 5) of α-dehydrotomatine **(A,E,I)**, α-tomatine **(B,F,J)** *m/z* intensities, *JRE4* **(C,G,K)**, and *GAME1* **(D,H,L)** measured at the nematodes’ invasion **(A-D)**, galling **(E-H)**, or reproduction **(I-L)** stages. Different letters above the boxplots indicate significant differences (*p* ≤ 0.05) in mean values between treatments, determined by Tukey's HSD test after ANOVA analysis.

We first analyzed the impact of *M. incognita* on the root concentration of α-dehydrotomatine and α-tomatine as well as on the expression of *JRE4* and *GAME1* genes throughout its infection cycle. *Meloidogyne incognita* root infection at the invasion and reproduction stages did not significantly affect the root level of α-dehydrotomatine and α-tomatine or the expression of *JRE4* and *GAME1* compared to controls (Figures 6A–D,I–L; blue vs. yellow boxplots; Supplementary Tables 7 and 13). When *M. incognita* was in the galling stage, its feeding increased the level of α-dehydrotomatine and α-tomatine and the expression of *JRE4* and *GAME1* compared to controls (Figures 6E–H; blue vs. yellow boxplots; Supplementary Tables 7 and 13).

We then assessed the systemic impact of *Ma. euphorbiae* leaf herbivory on the root defense expression. Leaf herbivory by *Ma. euphorbiae* did not affect the level of α-dehydrotomatine and α-tomatine, nor the expression of *JRE4* and *GAME1*, regardless of plant age (Figure 6; black vs. yellow boxplots; Supplementary Tables 7 and 13). These results indicate that leaf feeding by *Ma. euphorbiae* does not systemically alter the steroidal glycoalkaloids metabolism pathway in tomato roots.

Finally, we analyzed whether *Ma. euphorbiae* feeding systemically affects the root levels of steroidal glycoalkaloids and the expression patterns of GAME genes associated with *M. incognita* root infection. In general, the levels of α-dehydrotomatine and α-tomatine, and the expression of *JRE4* and *GAME1* of *M. incognita*-infected roots were similar to those in co-infected plants, regardless of the *M. incognita* infection cycle stage [Figure 6; red (MiMe) vs. blue (Mi) boxplots; Supplementary Tables 7 and 13]. Only in the case of *JRE4* expression, a higher expression level was found in the roots of co-infected plants compared to the roots of plants infected with *M. incognita* at the reproduction stage (Figure 6K; red vs. blue boxplot; Supplementary Tables 7 and 13). Overall, these results show that *Ma. euphorbiae* leaf herbivory has only a minor effect on root steroidal glycoalkaloid induction associated with *M. incognita* root infection.

### Impact of *Meloidogyne incognita* Root Infection on the Reproduction of *Macrosiphum euphorbiae*

We found an overall stronger effect of *M. incognita* root infection on the plant responses triggered by *Ma. euphorbiae* in leaves, compared to the reverse interaction (i.e., the reciprocal effect of *Ma. euphorbiae* feeding on root induced responses by *M. incognita* infection). Because of this, we next aimed to assess the impact of *M. incognita* root infection on the reproduction rate of *Ma. euphorbiae*. Similar to the defense response bioassays, we used the *M. incognita* infection cycle stages to time the performance bioassay. On plants without *M. incognita* root infection, we found that the numbers of nymphs produced on young and medium-age plants was significantly higher compared to those found on old (flowering) plants (Table 1). In plants that were challenged with *M. incognita*, we found a similar number of nymphs, at every nematode root infection stage compared to those observed in plants without *M. incognita* (Table 1).

**Table 1:** Number of nymphs produced by *Macrosiphum euphorbiae* adults on tomato plants.

Parameters	Source of variation	*M. incognita* root infection cycle stages		
	Mean ± SEM	Invasion	Galling	Reproduction
Control		12.6 ± 0.86^a^	14.2 ± 1.66^a^	4.0 ± 1.37^bc^
Mi		10.6 ± 1.03^ab^	15.2 ± 0.91^a^	2.8 ± 1.36^c^
Student *t*-test	*T*-value	0.9853	−0.3104	0.7530
	*Df*	8	8	8
	*p*-value	0.3533	0.7642	0.4731
Two-way ANOVA		*Df*	*F*	*p*
	Mi	1	0.284	0.599
	*T*	2	24.007	<0.001
	Mi^∗^*T*	2	0.425	0.659

*The number of Ma. euphorbiae nymphs produced were counted on tomato plants without root infection (Control) and with root-infection by Meloidogyne incognita (Mi). In co-infected plants, infestation with Ma. euphorbiae was performed at the nematodes’ invasion, galling, or reproduction stages. Data are means ± SEM (n = 5; per treatment). Two-way ANOVA (Df, F, and p-values) evaluated the effect of M. incognita life cycle stages on the Ma. euphorbiae reproduction. Student t-test (T-values, Df, and p-values are shown) tested the difference between means of the treatments per infection stage. Different lowercase superscript letters down column and along the rows after the means indicate significant differences (p ≤ 0.05) in mean values between treatments per life cycle stage, determined by Tukey's HSD test after ANOVA analysis. §; T: T-statistics, Df: degrees of freedom, F: statistics, p: probability value, Mi: Meloidogyne incognita, T: timepoint, Mi∗T: interaction between M. incognita and timepoint.*

## Discussion

Here, we used tomato as a model plant, to explore how root infection by *M. incognita* affects the leaf responses triggered by *Ma. euphorbiae*, and the reciprocal impact of leaf herbivory by *Ma. euphorbiae* on root responses induced by *M. incognita* infection in roots. Because root responses to *M. incognita* infection are tightly modulated during its infection cycle stages (Mbaluto et al., 2020), we studied the dynamics of the interaction between the induced plant responses by the two herbivores during the entire *M. incognita* root infection cycle. We show that root infection by *M. incognita* had mild systemic effects on phytohormones and steroidal glycoalkaloid responses triggered by *Ma. euphorbiae* locally on leaves. On the reverse, leaf-feeding by *Ma. euphorbiae* did not interfere systemically with the defense responses triggered by *M. incognita* locally in roots. In both interaction directions, the induction of defense responses occurred depending on the *M. incognita* root infection cycle stages. Collectively, our results indicate that root infection by *M. incognita* induces a strong effect in roots that is not overruled by AG *Ma. euphorbiae* feeding. They also demonstrate that the root infection cycle of *M. incognita* is an important factor influencing the dynamics of the interaction between the two herbivores.

We found that feeding by *Ma. euphorbiae* did not significantly affect phytohormonal signaling locally in leaves. In contrast to our results, several studies revealed that plants can activate the SA pathway upon attack by aphids, including *Ma. euphorbiae* (Mohase and van der Westhuizen, 2002; Chaman et al., 2003; Kuśnierczyk et al., 2008; Coppola et al., 2013). For instance, an increase in the expression of SA-responsive genes has been reported in *A. thaliana* upon the attack by *M. persicae* (Moran and Thompson, 2001) and by *Schizaphis graminum* on aphid-susceptible barley (Chaman et al., 2003). Moreover, the attack by *M. euphobiae*, *B. brassicae*, or *M. persicae* triggered the expression of both SA- and JA-responsive genes in *A. thaliana* and tomato plants (de Ilarduya et al., 2003; Kuśnierczyk et al., 2008; Coppola et al., 2013). Although we do not have a clear explanation for such apparent discrepancies with our results, the different outcomes may be partly explained by differences in the experimental set-ups, including the number and different stages of aphids or the duration of the experiments. Moreover, the differences in the studies can be due to the fact that piercing-sucking herbivores may antagonize defense responses to make the plant a more suitable host, depending on the system under investigation (Walling, 2008). Aphid salivary secretions contain effector proteins that may suppress defense responses (Hogenhout and Bos, 2011; Kettles and Kaloshian, 2016). In line with this, we found that *Ma. euphorbiae* infestation reduced the levels of the steroidal glycoalkaloids α-dehydrotomatine and α-tomatine and the expression of steroidal glycoalkaloid-related gene *JRE4* in leaves. *Solanum* alkaloids have a broad range of biological activity against insect herbivores, including aphids (Chowański et al., 2016). Thus, our results suggest the ability of *Ma. euphorbiae* to manipulate the secondary chemistry of the host plant to its benefit. Previous studies showed that aphids, including *Ma. euphorbiae* and *M. perciase*, can decrease secondary metabolites as well as trigger the downregulation of a set of alkaloid biosynthesis genes in tomato and *A. thaliana* (Mewis et al., 2012; Coppola et al., 2013). Interestingly, in this study, the aphid-triggered decrease in steroidal glycoalkaloids was specifically observed when the aphid fed on plants at the vegetative stage (young and medium-aged plants). By contrast, *Ma. euphorbiae* failed to counteract the steroidal glycoalkaloid-related responses in plants at the flowering stage (old plants). This indicates that plant age and ontogeny are important factors determining the ability of *Ma. euphorbiae* to modulate defense responses in tomato plants. In accordance to the inability of *Ma. euphorbiae* to suppress steroidal glycoalkaloid-related responses in flowering plants, we found that it performed worse when feeding on plants at the flowering stage (old plants), compared to plants in the vegetative stage. This is further evidence that the suppression of steroidal glycoalkaloid-related responses in local tissues can be important for aphid’s performance.

Whereas *Ma. euphorbiae* feeding did not induce phytohormonal responses locally in leaves, it systemically decreased the levels of JA, ABA, and IAA in roots, suggesting that this aphid might alter the allocation of defenses between roots and leaves. It has been previously demonstrated that aphids can reduce aliphatic glucosinolates in the roots. This led to a shift in the ratio of aliphatic and indole glucosinolates in systemic tissues, indicating that plants alter the allocation of defense compounds upon aphid attack (Kutyniok and Muller, 2012). Remarkably, the systemic impact of *Ma. euphorbiae* on root phytohormonal responses was only observed when it fed on flowering plants (old plants). In the same plants, we observed a trend for reduced levels of steroidal glycoalkaloids in roots. This suggests that plant age and ontogeny are also important factors influencing the systemic effect of *Ma. euphorbiae* leaf herbivory on root responses. Possibly, plants prioritize the allocation of defenses to reproductive tissues over vegetative tissues after herbivory (Chrétien et al., 2018). However, the ecological consequences of the decrease in the levels of phytohormones and glycoalkaloids triggered systemically by the aphids in the roots of the flowering tomato plants remain unclear.

Root infection by *M. incognita* triggered an increase of SA levels locally in roots throughout the entire infection cycle. Local accumulation of SA in roots upon the infection by different RKN species was found in several plant species, including *A. thaliana*, rice, and tomato (Branch et al., 2004; Hamamouch et al., 2011; Kumari et al., 2016; Guo and Ge, 2017). *Meloidogyne incognita* infection further led to an increase in ABA levels, specifically when it was at the reproduction stage. Increases in ABA are associated with increasing the susceptibility to *Meloidogyne* infection (Kyndt et al., 2017a). Therefore, we speculate that the increase in ABA levels triggered by *M. incognita* at the reproduction stage might be related to an enhancement of host susceptibility to the next generation of infective juveniles.

Besides the changes in phytohormone levels *M. incognita* infection also altered the steroidal glycoalkaloid response locally in roots. Indeed, specifically at the galling stage, *M. incognita* triggered an increase in the levels of the steroidal glycoalkaloids α-dehydrotomatine and α-tomatine and upregulated the expression of the steroidal glycoalkaloid related genes *JRE4* and *GAME1*. In agreement with our results, an increase in α-tomatine levels has been reported in tomato plants infected by *M. incognita* at the galling stage (Elliger et al., 1988). The induction of steroidal glycoalkaloids is associated with enhanced resistance to root infecting plant-parasitic nematodes (Wang et al., 2012; Jang et al., 2015). Therefore, the relevance of the increased steroidal glycoalkaloid levels in the present study remains unclear.

We further found that *M. incognita* root infection had just mild effects on the hormonally regulated pathways systemically in the leaves. Such a mild effect of root infecting plant-parasitic nematodes on systemic phytohormone signaling in AG tissues has been previously observed (Hamamouch et al., 2011; Kutyniok and Muller, 2012; Hoysted et al., 2017). On the other hand, *M. incognita* root infection reduced the levels of steroidal glycoalkaloids systemically in leaves, although this effect was significant just for α-dehydrotomatine in the invasion stage. Although it is unclear how systemic repression of host plant defenses can benefit nematode parasitism, some nematode effectors can suppress systemic signaling of defense responses in AG (Kyndt et al., 2014). These results indicate that *M. incognita* can cause subtle systemic changes in major defense compounds of tomato.

In co-infected plants, infection with *M. incognita*, in general, did not affect the leaf phytohormonal profile associated with *Ma. euphorbiae* leaf-feeding. However, the increase in SA levels triggered systemically in leaves by *M. incognita* infection at the reproduction stage was also evident in leaves of plants that were co-infected with both herbivores. This indicates that *Ma. euphorbiae* was unable to counteract SA signaling triggered systemically by *M. incognita* root infection. *M. incognita* infection further affected the steroidal glycoalkaloid-related responses triggered by *Ma. euphorbiae* feeding on plants at the vegetative stage. Indeed, the levels of α-dehydrotomatine and α-tomatine in leaves of co-infected plants were in between the levels found in controls and *Ma. euphorbiae* plants. This indicates that *M. incognita* infection counteracted, at least partially, the decrease in the levels of steroidal glycoalkaloids triggered by *Ma. euphorbiae*. Remarkably, these interactions did not affect the performance of the aphids. Previous studies show that SA levels can increase AG after root infection by plant-parasitic nematodes, but these changes differentially affect AG piercing-sucking insect herbivores. Guo and Ge (2017) reported an increase in SA levels in leaves of tomato plants infected by *M. incognita* in roots, which was concomitant with a reduction in the performance of whiteflies (*Bemisia tabaci*). On the other hand, Hoysted et al. (2017) found an increase in SA level in leaves of potato plants that were infected with *G. pallida* in roots, which correlated with the greater reproductive success of *M. persicae*. Taking together these studies, the variations in the findings can be attributed to differences in herbivores specialization and the plant system under investigation.

In roots, *Ma. euphorbiae* did not affect the phytohormonal profile associated with *M. incognita* root infection. The increased SA levels triggered by *M. incognita* throughout its infection cycle were still evident in the roots of co-infected plants. In analogy, *Ma. euphorbiae* did not interfere with the increased ABA levels triggered by *M. incognita* at the reproduction stage, even when *Ma. euphorbiae* infestation alone decreased the JA, ABA, and IAA levels systemically in roots. In line with this, *Ma. euphorbiae* feeding did not interfere with the increased levels of steroidal glycoalkaloids triggered by *M. incognita* at the galling stage. This further corroborates that the local effect of *M. incognita* determined the plant responses regardless of the later arriving herbivore *Ma. euphorbiae*. However, in our experimental design, the aphids were feeding for a limited time (24 h). Therefore, we cannot rule out a possible effect of *Ma. euphorbiae* on *M. incognita*-triggered plant responses nor on the performance of *M. incognita* at a later time points after aphid infestation.

In conclusion, we found that both *M. incognita* and *Ma. euphorbiae* triggered different local and systemic defense responses in tomato plants. When both herbivores co-occurred, *M. incognita* caused mild systemic effects on the induced plant responses to *Ma. euphorbiae* herbivory in leaves, which were not associated with changes in aphid’s performance. On the other hand, *M. incognita*-induced local root responses were not overruled by the systemic effect caused by *Ma. euphorbiae* leaf feeding, suggesting an asymmetrical interaction between *M. incognita* and *Ma. euphorbiae* when co-occurring in tomato plants.

## Data Availability Statement

The data underlying this study are published as open access at the iDiv Data Repository (https://idata.idiv.de/ddm/Data/ShowData/1876?version=0). The data can be cited as CMM, AMM, and NMvD (2021). Above and belowground systemic induced plant responses to root infection by root-knot nematode and leaf feeding by the potato aphid. iDiv Data Repository (https://doi.org/10.25829/idiv.1876-13-8394).

## Author Contributions

CMM, NMvD, and AMM conceived the research idea and designed the experiment. CMM and EMA performed the experiments. CMM, EMA, AMM, and KG processed the samples and analyzed the data. CMM conducted the literature search and wrote the initial manuscript with support of AMM and NMvD. All authors contributed to writing, reviewing, and approval of the final manuscript for submission.

### Conflict of Interest

The authors declare that the research was conducted in the absence of any commercial or financial relationships that could be construed as a potential conflict of interest.
