# Supplementary material for: Induced Local and Systemic Defense Responses in Tomato Underlying Interactions Between the Root-Knot Nematode Meloidogyne incognita and the Potato Aphid Macrosiphum euphorbiae
Source: Front Plant Sci. 2021 Apr 14;12:632212. doi: 10.3389/fpls.2021.632212 (PMC8081292; doi:10.3389/fpls.2021.632212)
Supplement: Supplementary file 1 [file Data_Sheet_1.PDF]

SUPPLEMENTARY MATERIAL

Supplementary Table 1. List of primers sequences used for qPCR.

Target gene	Target pathway	Primer sequences (5'-3')
Proteinase inhibitor II (PI II)[a]	Jasmonic acid (JA)	Fw: GAAAATCGTTAATTTATCCCAC<br>Rv: ACATACAAACTTTCCATCTTTA
Pathogen-related Protein 1 (PR1)[a]	Salicylic acid (SA)	Fw: GTGGGATCGGATTGATATCCT<br>Rv: CCTAAGCCACGATACCATGAA
Jasmonate-responsive ethylene response factor (ERF) 4 transcription factor (JRE4)[b]	Steroidal glycoalkaloids biosynthesis	Fw: TGTTTCCTCCGGTGTTACGG<br>Rv: CGATTTTTTCGAAACTCTTTCC
Glycoalkaloid metabolism 1 (GAME1)[b]		Fw: TTGCCGGATGTTCCATGATCG<br>Rv: CTAATGAAGAAACAGCGTCCTGG
SlEF X14449 (reference gene)[a]	Elongation factor-1α	Fw: GATTGGTGGTATTGGAACTGTC<br>Rv: AGCTTCGTGGTGCATCTC

Fw: forward, Rv: reverse

[a]Martínez-Medina, A., Fernández, I., Sánchez-Guzmán, M. J., Jung, S. C., Pascual, J. A., and Pozo, M. J. (2013). Deciphering the hormonal signalling network behind the systemic resistance induced by *Trichoderma harzianum* in tomato. *Front. Plant Sci.* 4, 1–12. doi:10.3389/fpls.2013.00206.

[b]Abdelkareem, A., Thagun, C., Nakayasu, M., Mizutani, M., Hashimoto, T., and Tsubasa Shoji (2017). Jasmonate-induced biosynthesis of steroidal glycoalkaloids depends on COI1 proteins in tomato. *Biochem. Biophys. Res. Commun.* 489, 206–210. doi:10.1016/j.bbrc.2017.05.132.

Supplementary Table 2. Results of three-way ANOVAs of the leaf concentration of jasmonyl-*L*-isoleucine (JA-*Ile*), salicylic acid (SA), abscisic acid (ABA) and indole-3-acetic acid (IAA) upon root infection by *Meloidogyne incognita* (Mi) and leaf infestation by *Macrosiphum euphorbiae* (Me), throughout the *M. incognita* infection cycle (time: T). Statistically significant effects are indicated in bold.

Hormone /	Model explanatory factor						
Source of variation	Mi	Me	T	Mi*Me	Mi*T	Me*T	Mi*Me*T
JA-*Ile*	n.s.	n.s.	n.s.	n.s.	n.s.	n.s.	n.s.
SA	**<0.001**	n.s.	**<0.001**	n.s.	**<0.001**	n.s.	n.s.
ABA	n.s.	n.s.	**<0.001**	n.s.	n.s.	n.s.	n.s.
IAA	**0.012**	**0.005**	**<0.001**	n.s.	n.s.	n.s.	n.s.

§: n.s. non-significant

Supplementary Table 3. Results of three-way ANOVAs of the leaf expression of *Proteinase inhibitor II (PI II)* and *Pathogenesis-related protein 1 (PR1)* and upon root infection by *Meloidogyne incognita* (Mi) and leaf infestation by *Macrosiphum euphorbiae* (Me), throughout the *M. incognita* root infection cycle (time: T). Statistically significant effects are indicated in bold.

Marker gene /	Model explanatory factor						
Source of variation	Mi	Me	T	Mi*Me	Mi*T	Me*T	Mi*Me*T
PI II	n.s.	**0.027**	**0.036**	n.s.	n.s.	n.s.	n.s.
PR1	n.s.	**0.041**	**0.018**	n.s.	n.s.	n.s.	n.s.

§: n.s. non-significant

Supplementary Table 4. Results of three-way ANOVAs of the leaf concentration of steroidal glycoalkaloids (*α*-dehydrotomatine and *α*-tomatine) and leaf expression of glycoalkaloid metabolism genes *(Jasmonate-responsive ethylene response factor (ERF) 4 transcription factor -JRE4* and *glycoalkaloid metabolism 1 -GAME1)* upon root infection by *Meloidogyne incognita* (Mi) and leaf infestation by *Macrosiphum euphorbiae* (Me), throughout the *M. incognita* root infection cycle (time: T). Statistically significant effects are indicated in bold.

Steroidal glycoalkaloids and GAME genes / Source of variation	Model explanatory factor						
	Mi	Me	T	Mi*Me	Mi*T	Me*T	Mi*Me*T
α-dehydrotomatine	n.s.	n.s.	**<0.001**	n.s.	n.s.	n.s.	n.s.
α-tomatine	n.s.	n.s.	**<0.001**	n.s.	n.s.	n.s.	n.s.
JRE4	**0.032**	**<0.001**	**<0.001**	n.s.	n.s.	n.s.	n.s.
GAME1	**0.072**	**0.044**	**<0.001**	n.s.	n.s.	n.s.	n.s.

§: n.s. non-significant

Supplementary Table 5. Results of three-way ANOVAs of the root concentration of jasmonic acid (JA), jasmonyl-*L*-isoleucine (JA-*Ile*), salicylic acid (SA), abscisic acid (ABA) and indole-3-acetic acid (IAA) upon root infection by *Meloidogyne incognita* (Mi) and leaf infestation by *Macrosiphum euphorbiae* (Me), throughout the *M. incognita* infection cycle (time: T). Statistically significant effects are indicated in bold.

Hormone /	Model explanatory factor						
Source of variation	Mi	Me	T	Mi*Me	Mi*T	Me*T	Mi*Me*T
JA	**<0.001**	n.s.	**<0.001**	n.s.	n.s.	n.s.	n.s.
JA-*Ile*	**<0.001**	n.s.	**<0.001**	n.s.	**0.024**	**0.024**	**0.020**
SA	**<0.001**	n.s.	**<0.001**	**0.035**	**<0.001**	n.s.	n.s.
ABA	**<0.001**	n.s.	**<0.001**	n.s.	**<0.001**	n.s.	**0.012**
IAA	n.s.	n.s.	**<0.001**	n.s.	n.s.	n.s.	n.s.

§: n.s. non-significant

Supplementary Table 6. Results of three-way ANOVAs of the root expression of *Proteinase inhibitor II (PI II)* and *Pathogenesis-related protein 1 (PR1)* and upon root infection by *Meloidogyne incognita* (Mi) and leaf infestation by *Macrosiphum euphorbiae* (Me), throughout the *M. incognita* root infection cycle (time: T). Statistically significant effects are indicated in bold.

Marker gene /	Model explanatory factor						
Source of variation	Mi	Me	T	Mi*Me	Mi*T	Me*T	Mi*Me*T
PI II	n.s.	**0.026**	**<0.001**	n.s.	n.s.	n.s.	**0.005**
PR1	n.s.	**0.047**	**<0.001**	n.s.	n.s.	**<0.001**	n.s.

§: n.s. non-significant

Supplementary Table 7. Results of three-way ANOVAs of the root concentration of steroidal glycoalkaloids (α-dehydrotomatine and α-tomatine) and root expression of glycoalkaloid metabolism genes *(Jasmonate-responsive ethylene response factor (ERF) 4 transcription factor -JRE4* and *glycoalkaloid metabolism 1 -GAME1)* upon root infection by *Meloidogyne incognita* (Mi) and leaf infestation by *Macrosiphum euphorbiae* (Me), throughout the *M. incognita* root infection cycle (time: T). Statistically significant effects are indicated in bold.

Steroidal glycoalkaloids and GAME genes / Source of variation	Model explanatory factor						
	Mi	Me	T	Mi*Me	Mi*T	Me*T	Mi*Me*T
α-dehydrotomatine	**<0.001**	n.s.	**<0.001**	n.s.	**0.006**	n.s.	n.s.
α-tomatine	**<0.001**	**0.035**	**<0.001**	n.s.	**0.009**	**0.013**	n.s.
JRE4	**0.032**	**<0.001**	**<0.001**	n.s.	n.s.	n.s.	n.s.
GAME1	**<0.001**	n.s.	**<0.001**	n.s.	**<0.001**	n.s.	n.s.

§: n.s. non-significant

Supplementary Table 8. Results of two-way ANOVAs of the leaf concentration of jasmonyl-*L*-isoleucine (JA-*Ile*), salicylic acid (SA), abscisic acid (ABA) and indole-3-acetic acid (IAA) upon root infection by *Meloidogyne incognita* (Mi) and leaf infestation by *Macrosiphum euphorbiae* (Me), at the different stages of nematode root infection (invasion, galling, or reproduction). Statistically significant effects are indicated in bold.

Hormone	Source of variation	Invasion			Galling			Reproduction		
		$Df_{(n,d)}$	F	P	$Df_{(n,d)}$	F	P	$Df_{(n,d)}$	F	P
JA-*Ile*	Mi	1,13	1.233	0.287	1,16	0.087	0.772	1,13	1.066	0.321
	Me	1,13	0.134	0.720	1,16	0.124	0.729	1,13	0.025	0.877
	Mi*Me	1,13	0.095	0.763	1,16	0.284	0.602	1,13	0.141	0.713
SA	Mi	1,11	1.904	0.195	1,16	3.849	0.067	1,16	88.855	**<0.00**
	Me	1,11	0.326	0.580	1,16	0.770	0.393	1,16	0.082	0.778
	Mi*Me	1,11	1.625	0.229	1,16	0.012	0.916	1,16	0.498	0.490
ABA	Mi	1,16	0.000	0.991	1,14	0.338	0.570	1,15	1.091	0.313
	Me	1,16	0.114	0.740	1,14	1.211	0.290	1,15	2.823	0.114
	Mi*Me	1,16	0.232	0.636	1,14	0.001	0.982	1,15	4.191	0.059
IAA	Mi	1,16	0.585	0.456	1,15	0.644	0.435	1,15	5.867	**0.029**
	Me	1,16	0.089	0.769	1,15	2.410	0.141	1,15	6.277	**0.024**
	Mi*Me	1,16	0.049	0.827	1,15	0.244	0.628	1,15	0.021	0.887

§: $Df_{(n,d)}$: degrees of freedom (numerator, and denominator), F: F test value, P: probability value

Supplementary Table 9. Results of two-way ANOVAs of the leaf expression of *Proteinase inhibitor II (PI II)* and *Pathogenesis-related protein 1 (PR1)* and upon root infection by *Meloidogyne incognita* (Mi) and leaf infestation by *Macrosiphum euphorbiae* (Me), at the different stages of nematode root infection (invasion, galling, or reproduction). Statistically significant effects are indicated in bold.

Marker genes	Source of variation	Invasion			Galling			Reproduction		
		$Df_{(n,d)}$	F	P	$Df_{(n,d)}$	F	P	$Df_{(n,d)}$	F	P
PI II	Mi	1,14	0.521	0.482	1,11	1.605	0.231	1,16	5.880	**0.028**
	Me	1,14	4.199	0.060	1,11	1.236	0.290	1,16	0.004	0.950
	Mi*Me	1,14	0.945	0.348	1,11	0.812	0.387	1,16	0.151	0.703
PR1	Mi	1,13	2.931	0.111	1,13	2.609	0.130	1,15	0.000	0.991
	Me	1,13	0.703	0.417	1,13	1.975	0.183	1,15	2.134	0.165
	Mi*Me	1,13	2.320	0.152	1,13	0.066	0.802	1,15	3.861	0.068

§: $Df_{(n,d)}$: degrees of freedom (numerator, and denominator); F: F test value, P: probability value

Supplementary Table 10. Results of two-way ANOVAs of the leaf concentration of steroidal glycoalkaloids (α-dehydrotomatine and α-tomatine) and leaf expression of glycoalkaloid metabolism genes *(Jasmonate-responsive ethylene response factor (ERF) 4 transcription factor -JRE4* and *glycoalkaloid metabolism 1 -GAME1)* upon root infection by *Meloidogyne incognita* (Mi) and leaf infestation by *Macrosiphum euphorbiae* (Me), at the different stages of nematode root infection (invasion, galling, or reproduction). Statistically significant effects are indicated in bold.

Steroidal glycoalkaloids and GAME genes	Source of variation	Invasion			Galling			Reproduction		
		$Df_{(n,d)}$	F	P	$Df_{(n,d)}$	F	P	$Df_{(n,d)}$	F	P
α-dehydro tomatine	Mi	1,14	0.028	0.870	1,14	0.065	0.802	1,13	0.189	0.671
	Me	1,14	2.396	0.144	1,14	3.530	0.081	1,13	0.926	0.354
	Mi*Me	1,14	16.572	**0.001**	1,14	0.619	0.445	1,13	1.561	0.234
α-tomatine	Mi	1,13	0.587	0.458	1,14	0.282	0.604	1,11	0.333	0.576
	Me	1,13	4.230	0.060	1,14	3.998	0.065	1,11	1.540	0.240
	Mi*Me	1,13	8.075	**0.014**	1,14	0.337	0.571	1,11	1.582	0.235
JRE4	Mi	1,14	3.336	0.089	1,13	1.618	0.226	1,12	1.102	0.315
	Me	1,14	17.169	**0.001**	1,13	4.829	**0.047**	1,12	7.142	**0.020**
	Mi*Me	1,14	1.328	0.269	1,13	0.002	0.967	1,12	1.249	0.286
GAME1	Mi	1,12	1.659	0.222	1,13	2.976	0.108	1,14	0.553	0.469
	Me	1,12	1.609	0.229	1,13	0.497	0.493	1,14	3.312	0.090
	Mi*Me	1,12	0.312	0.587	1,13	1.487	0.244	1,14	0.030	0.866

§: $Df_{(n,d)}$: degrees of freedom (numerator, and denominator); F: F test value, P: probability value

Supplementary Table 11. Results of two-way ANOVAs of the root concentration of jasmonic acid (JA), jasmonyl-*L*-isoleucine (JA-*Ile*), salicylic acid (SA), abscisic acid (ABA), and indole-3-acetic acid (IAA) upon root infection by *Meloidogyne incognita* (Mi) and leaf infestation by *Macrosiphum euphorbiae* (Me), at the different stages of nematode root infection (invasion, galling, or reproduction). Statistically significant effects are indicated in bold

Hormone	Source of variation	Invasion			Galling			Reproduction		
		$Df_{(n,d)}$	F	P	$Df_{(n,d)}$	F	P	$Df_{(n,d)}$	F	P
JA	Mi	1,14	6.113	**0.027**	1,14	3.994	0.066	1,13	8.651	**0.012**
	Me	1,14	3.668	0.076	1,14	0.105	0.751	1,13	0.675	0.426
	Mi*Me	1,14	0.049	0.829	1,14	0.588	0.456	1,13	11.338	**0.005**
JA-*Ile*	Mi	1,15	0.530	0.478	1,13	13.467	**0.001**	1,14	4.633	**0.049**
	Me	1,15	0.028	0.869	1,13	3.288	0.093	1,14	3.673	0.076
	Mi*Me	1,15	0.033	0.858	1,13	7.833	**0.015**	1,14	0.127	0.727
SA	Mi	1,14	15.285	**0.002**	1,14	25.369	**<0.00**	1,12	245.720	**<0.00**
	Me	1,14	0.157	0.698	1,14	2.498	0.136	1,12	0.328	0.578
	Mi*Me	1,14	0.732	0.407	1,14	2.543	0.133	1,12	0.534	0.479
ABA	Mi	1,13	8.455	**0.012**	1,12	1.499	0.244	1,16	303.817	**<0.00**
	Me	1,13	1.687	0.217	1,12	2.095	0.173	1,16	0.7011	0.415
	Mi*Me	1,13	2.699	0.124	1,12	1.368	0.265	1,16	21.817	**<0.00**
IAA	Mi	1,15	0.059	0.811	1,16	9.552	**0.007**	1,14	11.783	**0.004**
	Me	1,15	0.228	0.640	1,16	1.836	0.194	1,14	12.072	**0.004**
	Mi*Me	1,15	1.631	0.221	1,16	0.643	0.434	1,14	0.933	0.350

§: $Df_{(n,d)}$: degrees of freedom (numerator, and denominator); F: F test value, P: probability value

Supplementary Table 12. Results of two-way ANOVAs of the root expression of *Proteinase inhibitor II (PI II)* and *Pathogenesis-related protein 1 (PR1)* and upon root infection by *Meloidogyne incognita* (Mi) and leaf infestation by *Macrosiphum euphorbiae* (Me), at the different stages of nematode root infection (invasion, galling, or reproduction). Statistically significant effects are indicated in bold

Marker genes	Source of variation	Invasion			Galling			Reproduction		
		$Df_{(n,d)}$	F	P	$Df_{(n,d)}$	F	P	$Df_{(n,d)}$	F	P
PI II	Mi	1,14	2.281	0.153	1,15	4.921	**0.042**	1,11	0.026	0.874
	Me	1,14	2.811	0.116	1,15	10.319	**0.006**	1,11	0.362	0.560
	Mi*Me	1,14	2.585	0.130	1,15	2.107	0.167	1,11	4.651	0.054
PR1	Mi	1,14	1.223	0.288	1,13	5.375	**0.037**	1,13	0.096	0.762
	Me	1,14	2.142	0.165	1,13	6.000	**0.029**	1,13	8.172	**0.013**
	Mi*Me	1,14	0.907	0.357	1,13	1.136	0.306	1,13	0.403	0.537

§: $Df_{(n,d)}$: degrees of freedom (numerator, and denominator); F: F test value, P: probability value

Supplementary Table 13. Results of two-way ANOVAs of the root concentration of steroidal glycoalkaloids (α-dehydrotomatine and α-tomatine) and root expression of glycoalkaloid metabolism genes *(Jasmonate-responsive ethylene response factor (ERF) 4 transcription factor -JRE4* and *glycoalkaloid metabolism 1 -GAME1)* upon root infection by *Meloidogyne incognita* (Mi) and leaf infestation by *Macrosiphum euphorbiae* (Me), at the different stages of nematode root infection (invasion, galling, or reproduction). Statistically significant effects are indicated in bold.

Steroidal glycoalkaloids and GAME genes	Source of variation	Invasion			Galling			Reproduction		
		$Df_{(n,d)}$	F	P	$Df_{(n,d)}$	F	P	$Df_{(n,d)}$	F	P
α-dehydro tomatine	Mi	1,15	0.624	0.442	1,13	8.409	**0.012**	1,14	42.050	**<0.00**
	Me	1,15	0.602	0.450	1,13	0.148	0.707	1,14	8.481	**0.011**
	Mi*Me	1,15	2.073	0.170	1,13	6.270	**0.026**	1,14	1.933	0.186
α-*t*omatine	Mi	1,15	7.535	**0.015**	1,14	45.405	**<0.00**	1,15	10.177	**0.006**
	Me	1,15	0.514	0.485	1,14	3.716	0.075	1,15	5.734	**0.030**
	Mi*Me	1,15	0.508	0.487	1,14	4.410	0.054	1,15	1.260	0.279
JRE4	Mi	1,14	0.094	0.764	1,13	11.526	**0.005**	1,13	3.854	0.071
	Me	1,14	2.768	0.118	1,13	0.106	0.750	1,13	1.084	0.317
	Mi*Me	1,14	0.263	0.616	1,13	0.678	0.425	1,13	13.428	**0.003**
GAME1	Mi	1,14	5.685	**0.032**	1,12	69.557	**<0.00**	1,13	31.351	**<0.00**
	Me	1,14	0.031	0.863	1,12	3.527	0.085	1,13	0.265	0.616
	Mi*Me	1,14	0.190	0.670	1,12	0.770	0.397	1,13	2.072	0.174

§: $Df_{(n,d)}$: degrees of freedom (numerator, and denominator); F: F test value, P: probability value
